# Auto-Regulation Method vs. Fixed-Loading Method in Maximum Strength Training for Athletes: A Systematic Review and Meta-Analysis

**DOI:** 10.3389/fphys.2021.651112

**Published:** 2021-03-12

**Authors:** Xing Zhang, Hansen Li, Shilin Bi, Yong Luo, Yang Cao, Guodong Zhang

**Affiliations:** ^1^Department of Basketball and Volleyball, Chengdu Sport University, Chengdu, China; ^2^Key Lab of Physical Fitness Evaluation and Motor Function Monitoring of General Administration of Sports of China, College of Physical Education, Institute of Sports Science, Southwest University, Chongqing, China; ^3^National Institute of Education, Nanyang Technological University, Singapore, Singapore; ^4^Clinical Epidemiology and Biostatistics, School of Medical Sciences, Örebro University, Örebro, Sweden; ^5^Unit of Integrative Epidemiology, Institute of Environmental Medicine, Karolinska Institutet, Stockholm, Sweden

**Keywords:** auto-regulation method, fixed-loading method, maximum strength, training, athlete, meta-analysis

## Abstract

The auto-regulation method is a rising training strategy to improve strength and motor performance, and the Autoregulatory Progressive Resistance Exercise (APRE), Rating of Perceived Exertion program (RPE), and Velocity-Based Training (VBT) are the three common auto-regulation programs. However, whether the auto-regulation method is more effective than the traditional strength training (the fixed-loading method) in maximum strength training is still unclear. The present study searched the Pubmed, SPORTDiscus, Web of Science, Embase, EBSCO, Cochrane, CNKI, and CQVIP databases, and included eight related studies published between 2010 and 2020, with a total of 166 subjects including division 1 college players and athletes with at least 1-year training history, and interventions ranging from 5 to 10 weeks. A meta-analysis was performed to check the difference between the two training methods, and analyzed the differences in the existing auto-regulation programs' effectiveness. The overall results showed that the auto-regulation method was more effective than the fixed-loading method in maximum strength training (effect size = 0.64; *P* < 0.001; *I*^2^ = 0%). In specific, the pooled results in subgroup analysis indicated that the auto-regulation method may effectively improve the strength performance in squat (effect size = 4.64; *P* < 0.05; *I*^2^ = 54%) and bench press (effect size = 3.21; *P* < 0.05; *I*^2^ = 62%). Greater benefits of the auto-regulation method on strength improvement could be achieved in an 8-week or even shorter training (effect size = 0.87; *P* < 0.001; *I*^2^ = 0%) compared with those of 8–10 weeks (effect size = 0.32; *P* < 0.001; *I*^2^ = 0%). The APRE is the most effective training program among the three auto-regulation programs (effect size = 0.78; *P* < 0.001; *I*^2^ = 0%). In conclusion, the auto-regulation method could be more effective than the fixed-loading method in maximum strength training. The APRE is a convenient and effective training program that may be considered a practical training program to replace traditional training in athletes.

## Introduction

The maximum strength is known to play a key role in improving and maintaining sports performance, including increased speed (Ronnestad et al., 2008; Chelly et al., 2009; Comfort et al., 2012; Styles et al., 2016), agility (Spiteri et al., 2013, 2015), and explosive strength (HÄKkukinen et al., 1985; Van Cutsem et al., 1998; Aagaard et al., 2002; Chelly et al., 2009; Andersen et al., 2010), and is even conducive to develop motor skills (Suchomel et al., 2016). Besides, the developed maximum strength has also been associated with effective protection in body structures such as bones, ligaments, and tendons, which can further reduce risk in athletic injury and prolong the athletic career (Fleck and Falkel, 1986; Radin, 1986; Stone, 1988; Lehnhard et al., 1996; Lauersen et al., 2014). Therefore, to effectively develop the maximum strength, many attempts have been made to find out a better training method (Materko et al., 2010; Shalfawi and Kjellstadli, 2018).

In the past decades, traditional strength training seems to be the effective method in improving maximum strength, which refers to various training programs designed based on the individual's strength limitation, usually known as the 1RM (One Repetition Maximum) in strength events. Briefly, the working load of a fixed-loading method, such as the training intensity and volume, is designed and fixed before the training gets started, where the name “fixed-loading” of the traditional strength training comes from. In periodical training, the fixed-loading method needs to be performed with an accurate estimation of the supercompensation to achieve effective progress. Due to numerous successful cases, the fixed-loading method has been considered as the best strategy in strength training for a very long time, and has been applied in different sports and people with a varied athletic ability (Rhea et al., 2002; Sander et al., 2013).

However, though the importance of the fixed-loading method is indisputable, it also has some disadvantages. For instance, due to the diurnal variation of the physiological index, the maximum strength can change up to 10–20% (Poliquin, 1988), and other factors can also affect the sports performance, such as sleeping conditions, warmup programs, and sports supplements intake (Warren et al., 2009; Amiri-Khorasani and René, 2014; de Salles Painelli et al., 2014; Baxter et al., 2016; Abbott et al., 2020; Patterson et al., 2020), which makes it very hard to choose an appropriate working load according to an athlete's body condition (Kraemer and Fleck, 2007), thus the long-term development of strength is limited. Consequently, the uncertainties in training may result in overtraining or inadequate training, and followed by injury or degeneration in training (Poliquin, 1988).

In response, a flexible and adjustable strength training method, known as the auto-regulation method, was therefore developed to address this problem. This method aims to monitor and evaluate whether the working load is reasonable according to the specific performance of athletes. Besides, by regulating the working load (usually training reps and weights), athletes can receive proper training that in accord with their real-time conditions, and thus obtain optimal progress (Flanagan and Jovanovic, 2014). At present, the auto-regulation method mainly includes three important programs as follows:

(1) The Autoregulatory Progressive Resistance Exercise (APRE) is a program regulated based on the completed reps (Mann, 2011). The APRE program requires athletes to determine the training intensity (weight) of the first and the second sets in advance, and further adjust the training weight of the fourth set according to the completed reps of the third set. If the third set's completed reps are more than the target reps, the weight will be increased. Otherwise, the weight will be reduced.

(2) The Rating of Perceived Exertion program (RPE) is regulated using various RPE measuring scales (Helms et al., 2016). Among the scales, the Borg CR10 Scale (Shariat et al., 2018) and the OMNI-RES Scale (Robertson et al., 2003) were frequently used in the RPE program. The former scale is evaluated by a 10-point Likert scale, while the latter one uses an 11-point Likert scale. For the two scales, a higher score indicates more difficulty in finishing one rep. For example, the RPE 9 means an arduous attempt but still one more rep can be done, and the RPE10 means extremely hard and another rep is impossible.

(3) The Velocity-Based Training (VBT) is a program regulated based on the movement speed during the training (Flanagan and Jovanovic, 2014). This training program highly relies on the speed detector, and linear position sensors or wearable devices are usually employed in the strength training to monitor athletes' movement and provide feedback to regulate the working load. Besides, different training purposes require various speeds. For example, a speed below 0.5 m/s is considered effective to develop maximum strength (Izquierdo et al., 2006; Jidovtseff et al., 2009; Jandačka and Beremlijski, 2011). During the training, if the movement speed exceeds the speed range, the load will be increased; otherwise, the load will be reduced or the training will be terminated.

Nowadays, many studies have demonstrated the advantages of the auto-regulation method over the fixed-loading method. For instance, the auto-regulation method contributes to more significant improvements in strength endurance (Mann et al., 2010), explosive strength (Orange et al., 2019b; Dorrell et al., 2020), and speed (Orange et al., 2019a). However, the difference in maximum strength improvement between the auto-regulation method and the fixed-loading method is still unclear. Though some researchers believe that the auto-regulation method is superior to the fixed-loading method in improving the maximum strength (Mann et al., 2010; Mann, 2011; Graham and Cleather, 2019), some other evidence indicates no significant difference between the two training methods (Fisher, 2016; Helms et al., 2018; Patroklos et al., 2018). Therefore, this systematic review and meta-analysis aimed to:

(1) examine the difference between the two training methods;
(2) reveal their functions in different training events and interventions;
(3) quantify the differences among the APRE, RPE, and VBT programs in maximum strength training by synthesizing evidence from current published studies.

## Materials and Methods

### Selection Criteria

The following PICOS criteria for inclusion and exclusion of studies in the present study were:

- P (population): sportspeople who have strength training history for at least one year;
- I (intervention): using auto-regulation methods as intervention;
- C (comparison): using the fixed-loading method as control;
- O (outcomes): the 1RM was measured in the training events;
- S (study design): study design considered randomized-controlled trials (RCTs), cohort studies, and comparative studies that evaluated the effects of the auto-regulation methods in maximum strength training.

Meanwhile, the criteria for exclusion were:

- The study was not aimed to improve the maximum strength;
- The sample size did not meet the requirement of meta-analysis.

### Data Sources

A systematical search was conducted in both English and Chinese databases, including Pubmed, SPORTDiscus, Web of Science (all database), Embase, EBSCO (all database), Cochrance, CNKI (in Chinese), and CQVIP (in Chinese). Besides, to include more potential studies, we also screened the references in the preliminarily identified articles and included the related ones.

### Searching Strategy

Databases were searched from the inception of the databases up to October 8, 2020. The detailed strategy was listed in Table 1, demonstrated in Pubmed searching style, and the other databases were searched using the same strategy.

**Table 1:** Searching strategy for the study inclusion.

**Steps**	**Searching command**	**Field**
#1	Ratings of perceived exertion OR ratings of exertion OR perceived exertion OR RPE OR repetitions in reserve OR RIR OR autoregulation OR auto-regulation OR VBT OR Velocity Based OR APRE	Title
#2	Power training OR strength building OR strength training OR weight work OR resistance exercise OR work against resistance OR powerlifting OR weight lifting OR muscular strength OR 1RM strength	Tile or Abstract
#3	#1 AND # 2 AND	

### Literature Screening and Data Extraction

Literature screening was performed by two authors (XZ and HL) independently. The discrepancies were resolved by discussion, or referred to a third author (SB) for opinions. Data extracted include article title, author name, publication year, study design, participant profile, sample size, interventions, intervention measures, control measures, measurements, and outcomes. The number of study participants and means and standard deviations (SD) of 1RM tested before and after intervention were extracted from the articles and included in the meta-analysis. The SD was calculated using the reported standard error and the sample size if it was not directly available in an article.

### Quality Assessment

The PEDro scale (Physiotherapy Evidence-Based Database) was employed in the present study to assess the methodological quality of the included studies. The PEDro, which contains 11 items, has high reliability and validity according to the previous studies (Maher et al., 2003; de Morton, 2009). The items are scored as Yes (1 point), No (0 point), and Don't know (0 point). The first item (methodological item) will be demonstrated but not included in the total score, while the complete assessment takes into account the scores of 2–11 items, and the overall qualities are assessed as excellent (9–10 points), good (6–8 points), fair (4–5 points), and poor (<4 points) (Maher et al., 2003; PEDro Scale Physiotherapy Evidence Database, 2010). The assessment was independently carried out by two authors (HSL, ZX), and disagreements were discussed with the third author (SB) until consensus was reached.

### Statistical Analysis

The software Reviewer manager 5.3 was employed for the synthesis of the data. Heterogeneity was assessed using the standard *I*^2^ index and the chi-square tests. *I*^2^ values of 25, 50, and 75% were interpreted as representing small, moderate, and high levels of heterogeneity (Higgins et al., 2003). The random-effects model was employed for data synthesis if there was a high heterogeneity or inconsistency (*I*^2^ > 50%), otherwise, the fixed-effects model was performed instead. The standardized mean difference (SMD) was used to present the overall synthesis outcomes due to the different training items, while the mean difference (MD) was used to demonstrate the synthesis outcomes in subgroups. A sensitivity analysis was conducted via the leave-one-out method to identify the source of the heterogeneity and further check the stability and reliability of the results.

## Results

### Selection of Studies

**Figure 1:**
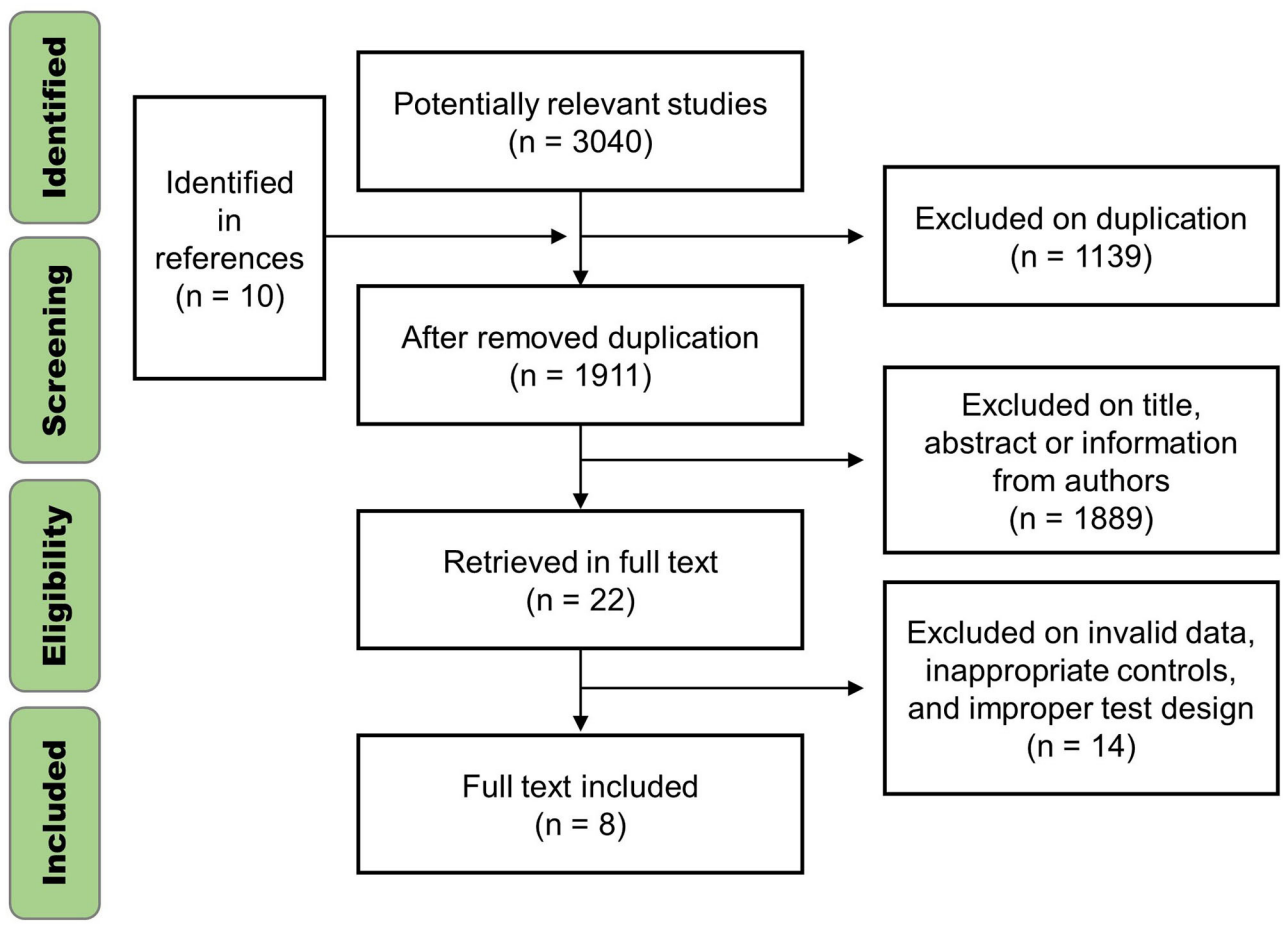
Flow diagram of screening and selection of studies.

In total, 3,040 studies were found from the searching, 3,018 were excluded due to duplication and screening by title and abstract, and 13 were excluded for inappropriate controls. An identified study with 31 subjects and a 12-week intervention was also excluded for data unavailability. As a result, eight studies (seven English studies and one Chinese study), which resulted in 17 reports, were included after the screening (Figure 1). The studies were published between 2010 and 2020. Three studies used the APRE program (Mann et al., 2010; Mann, 2011; Weber, 2015), two used the RPE program (Helms et al., 2018; Patroklos et al., 2018), and three used the VBT program (Fisher, 2016; Singh, 2016; Zhihui, 2020). The control groups used various fixed-loading programs, seven studies used the linear periodization programs (Mann et al., 2010; Mann, 2011; Weber, 2015; Fisher, 2016; Singh, 2016; Helms et al., 2018; Patroklos et al., 2018), one study used the fixed load of 75% 1RM (Zhihui, 2020). In terms of maximum strength measurement, three studies with 49 subjects used the direct test (attempts and failure) (Helms et al., 2018; Patroklos et al., 2018; Zhihui, 2020), and the rest five studies with 117 subjects evaluated the maximum strength via 1RM formula. A total of 166 subjects (151 males and 15 females) were included. All the subjects were young experienced athletes (training years > 1 year), and underwent different intervention durations of 5–10 weeks. Five reports indicated that the auto-regulation methods were more effective in maximum strength training than the fixed-loading method, while no significant difference was reported in the rest of the reports, or no statistical test was performed. We conducted the *t*-test if the data were available, but no statistically significant difference was found at the baseline. The detailed information of the included studies is shown in Table 2.

**Table 2:** Characteristics of studies included in the present study.

**Authors**	**Intervention**	**Design**	**1RM measurement**	**Gender**	**Intervention group**				**Control group**				**Results (superior method)**
					**Subject**	**Training year**	**Age**	**Training program**	**Subject**	**Training year**	**age**	**Training program**	
Mann et al. (2010)	6 weeks	Non-RCT	ES	Male	12	2.9 ± 0.7	20.2 ± 1.0	Bench press (APRE)	11	2.43 ± 0.7	20.3± 1.6	Bench press (Fixed)	Auto
								Squat (APRE)				Squat (Fixed)	*P* = 0.05
Weber (2015)	8 weeks	RCT	ES	Male	9	9.9 ± 3.4	20.4 ± 1.6	Squat (APRE)	9	10.3 ± 2.6	20.0 ± 1.1	Squat (Fixed)	n. s.
								Bench press (APRE)				Bench press (Fixed)	Auto
								Right grip strength (APRE)				Right grip strength (Fixed)	n. s.
								Left grip strength (APRE)				Left grip strength (Fixed)	n. s.
Mann (2011)	6 weeks	Non-RCT	ES	Male	32	Division 1 college	19.62	Bench press (APRE)	25	Division 1 college	19.13	Bench press (Fixed)	Auto
						player		Squat (APRE)		player		Squat (Fixed)	Auto
								Clean (APRE)				Clean (Fixed)	Auto
Helms et al. (2018)	8 weeks	MPD	DT	Male	10	>2	20.9 ± 1.4	Squat (RPE)	11	> 2	23.8 ± 4.2	Squat (Fixed)	n. s.
								Bench press (RPE)				Bench press (Fixed)	n. s.
Patroklos et al. (2018)	10 weeks	MPD	DT	Male	5	>2	27 ± 6	Squat (RPE)	3	> 2	27 ± 6	Squat (Fixed)	n. s.
								Bench press (RPE)				Bench press (Fixed)	n. s.
								Deadlift (RPE)				Deadlift (Fixed)	n. s.
Zhihui (2020)	8 weeks	RCT	DT	Male	10	3.20 ± 0.42	20.10 ± 0.88	Squat (VBT)	10	3.20 ± 0.42	20.10 ± 0.88	Squat (fixed)	n. s.
Singh (2016)	5 weeks	RCT	ES	Male	2	>2	22.2 ± 1.3	Bench press (VBT)	2	> 2	22.2 ± 1.3	Bench press (Fixed)	n. s.
Fisher (2016)	6 weeks	RCT	ES	Female	8	>1	20.00 ± 0.9	Bench press (VBT)	7	> 1	20.67 ± 0.9	Bench press (Fixed)	n. s.

*RCT, randomized controlled trails; MPD, matched-pairs design; Auto, auto-regulation method; n.s., non-significant difference; APRE, Autoregulatory Progressive Resistance Exercise; RPE, Rating of Perceived Exertion program; VBT, Velocity-Based Training; Fixed, fixed-loading programs; DT, direct test for 1RM; ES, 1RM was estimated via formula*.

### Quality of the Included Studies

Among the eight included studies, four studies used an RCT design, two used a matched-pairs design, and two used a non-RCT design. Three studies were assessed as good quality, and five as fair quality according to the PEDro scale (Table 3). All the eight studies lacked blinding, including blinding for subjects, therapists, and assessors.

**Table 3:** Quality assessment of the included studies.

**Study**	**PEDro item**												
	**1**	**2**	**3**	**4**	**5**	**6**	**7**	**8**	**9**	**10**	**11**	**Total**	**Assessment**
Mann et al. (2010)	Yes							1	1	1	1	4	Fair
Weber (2015)	No	1		1				1	1	1	1	6	Good
Mann (2011)	No			1				1	1	1	1	5	Fair
Helms et al. (2018)	Yes			1				1	1	1	1	5	Fair
Patroklos et al. (2018)	Yes			1					1	1	1	4	Fair
Zhihui (2020)	Yes	1		1				1	1	1	1	6	Good
Singh (2016)	Yes	1		1					1	1	1	5	Fair
Fisher (2016)	Yes	1		1				1	1	1	1	6	Good

*Items 1. Eligibility criteria were specified. 2. Subjects were randomly allocated to groups (in a crossover study, subjects were randomly allocated an order in which treatments were received). 3. Allocation was concealed. 4. The groups were similar at baseline regarding the most important prognostic indicators. 5. There was blinding of all subjects. 6. There was blinding of all therapists who administered the therapy. 7. There was blinding of all assessors who measured at least one key outcome. 8. Measures of at least one key outcome were obtained from more than 85% of the subjects initially allocated to groups. 9. All subjects for whom outcome measures were available received the treatment or control condition as allocated, or where this was not the case, data for at least one key outcome was analyzed by “intention to treat.” 10. The results of between-group statistical comparisons are reported for at least one key outcome. 11. The study provides both point measures and measures of variability for at least one key outcome*.

### Synthesis of Results

**Figure 2:**
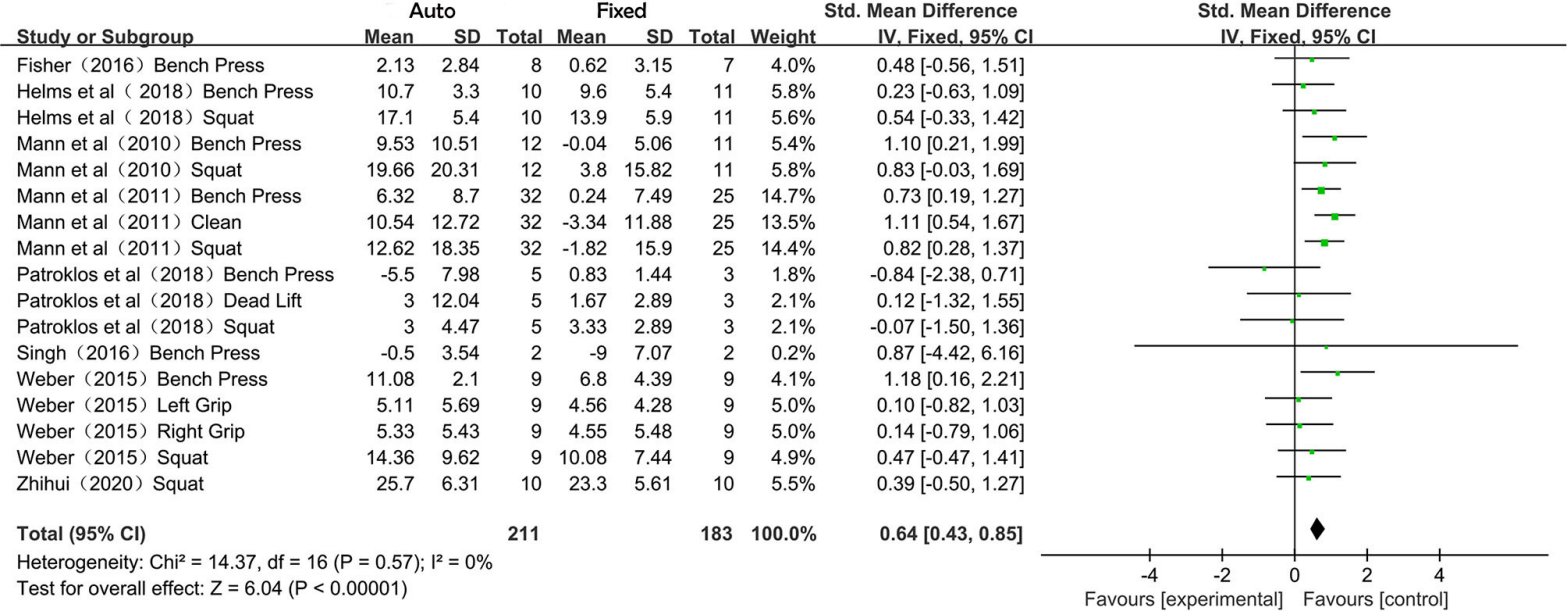
Forest plot referred to the meta-analysis for the overall effect of the auto-regulation method.

As no heterogeneity was observed among the 17 included reports (*I*^2^ = 0%), the synthesis was conducted using the fixed-effects model, and pooled results were presented in standard mean difference (SMD). The overall effect size was 0.64 (95% CI 0.43–0.85; *P* < 0.001) (Figure 2).

**Figure 3:**
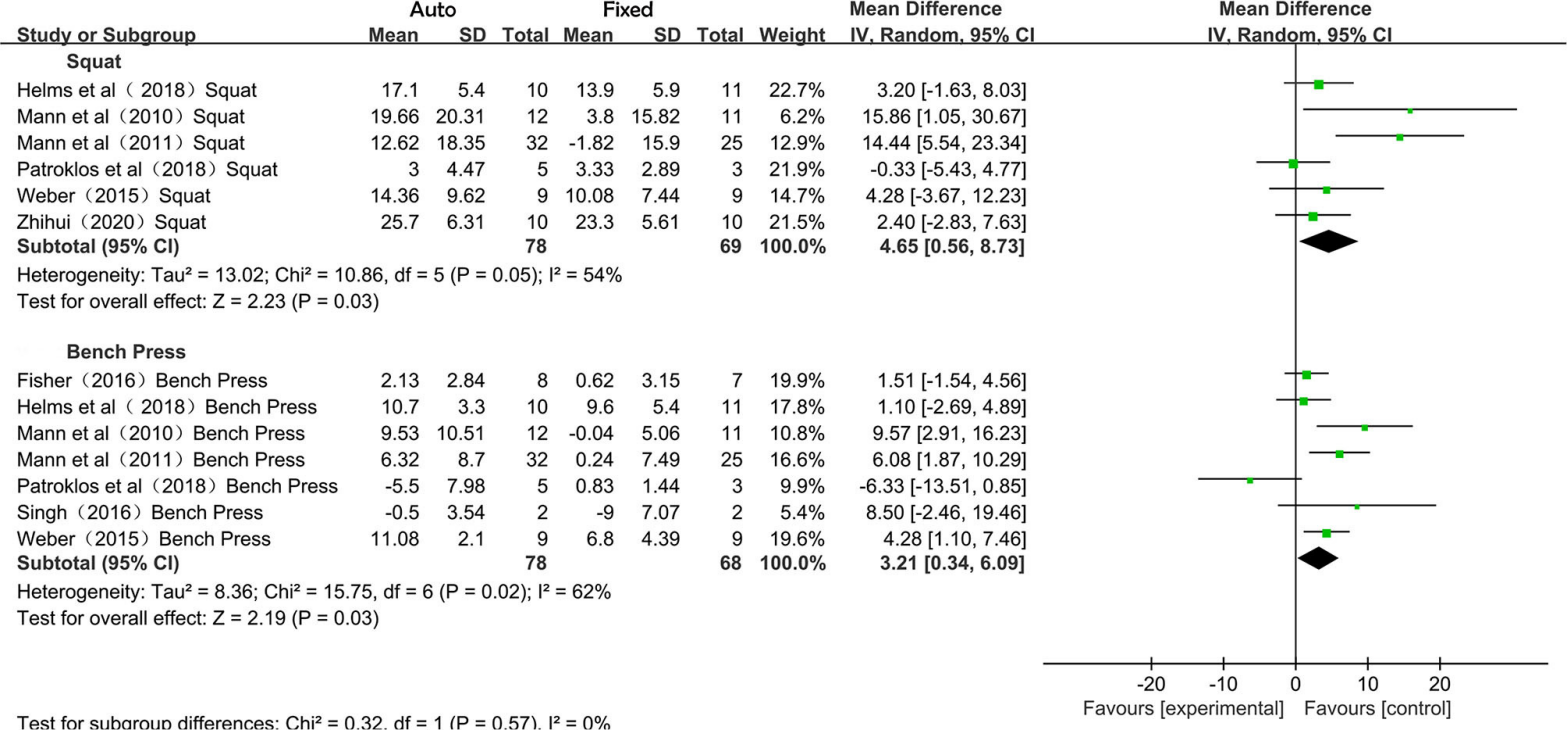
Forest plot referred to the effects of auto-regulation in strength training for squat and bench press.

Squat and bench press are the two main training events in the included studies (the other events were only investigated in one or two studies). Therefore, the subgroup analysis was performed to check the effects of auto-regulation methods on different events. As the 1RM was tested for squat and bench press in the included studies, the pooled results were presented in mean difference (MD), and due to medium to high heterogeneity was observed in the synthesis of both subgroups (*I*^2^ > 50%), the random-effects model was employed, and the overall effects of squat and bench press were 4.65 (95% CI 0.56–8.73; *P* < 0.05) and 3.21 (95% CI 0.34–6.09; *P* < 0.05), respectively (Figure 3). No statistically significant difference was found in the overall effects between the two training events (*P* = 0.57).

**Figure 4:**
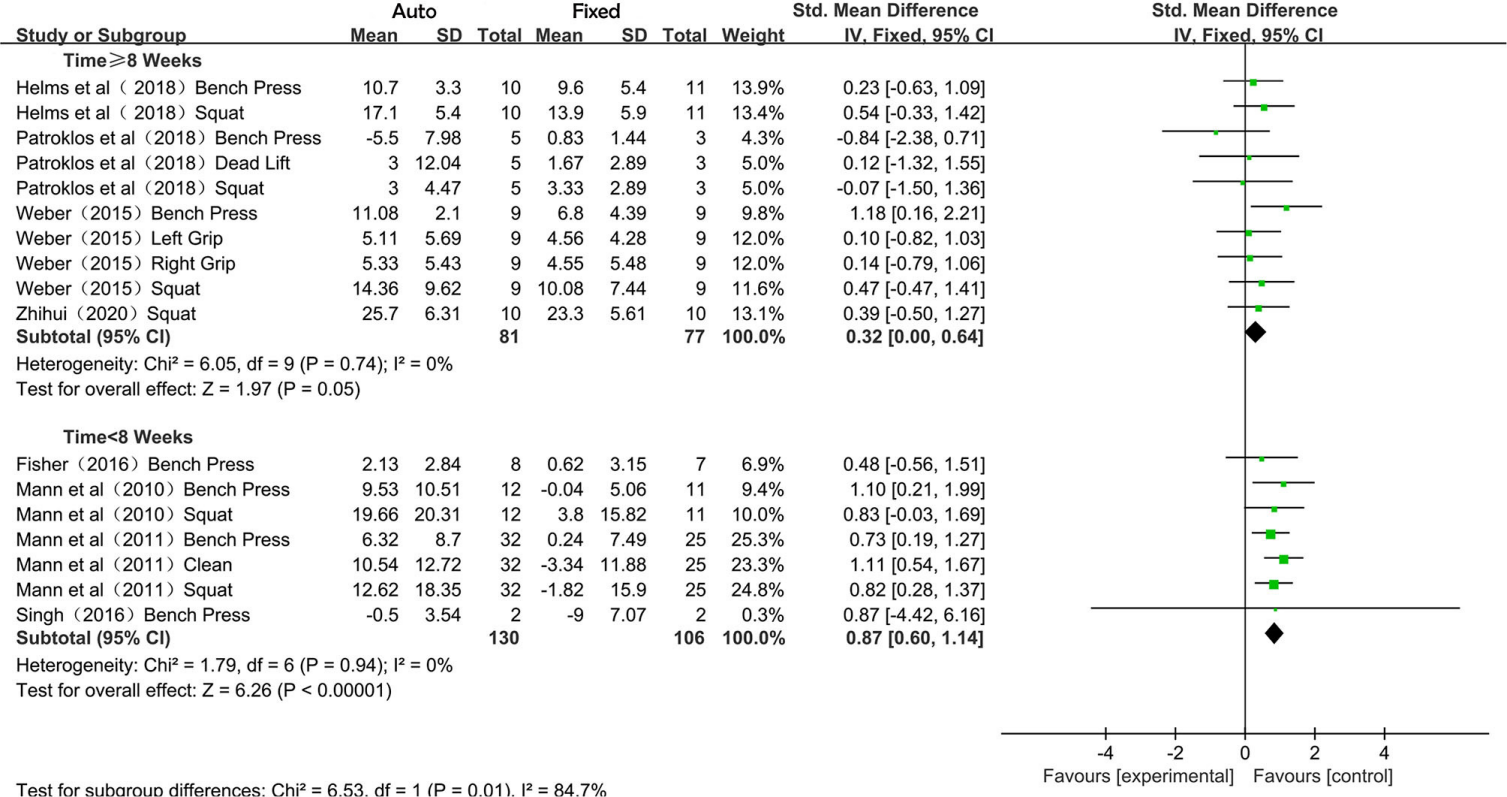
Forest plot referred to the effects of auto-regulation effects of different interventions in maximum strength training (5–7 vs. 8–10 weeks).

Due to the varied duration of intervention, the included studies were divided into short-term intervention (<8 weeks) and medium-term intervention (≥8 weeks). The subgroup analysis was carried out and the pooled results were presented in SMD (Figure 4). As no heterogeneity was observed in each of the subgroup synthesis (*I*^2^ = 0%), the fixed-effects model was employed, and the overall effects of the short-term and long-term intervention were 0.32 (95% CI 0.00–0.64; *P* = 0.05) and 0.87 (95% CI 0.60–1.14; *P* < 0.001). A statistically significant difference was found in the overall effects between the subgroups (*P* = 0.01).

**Figure 5:**
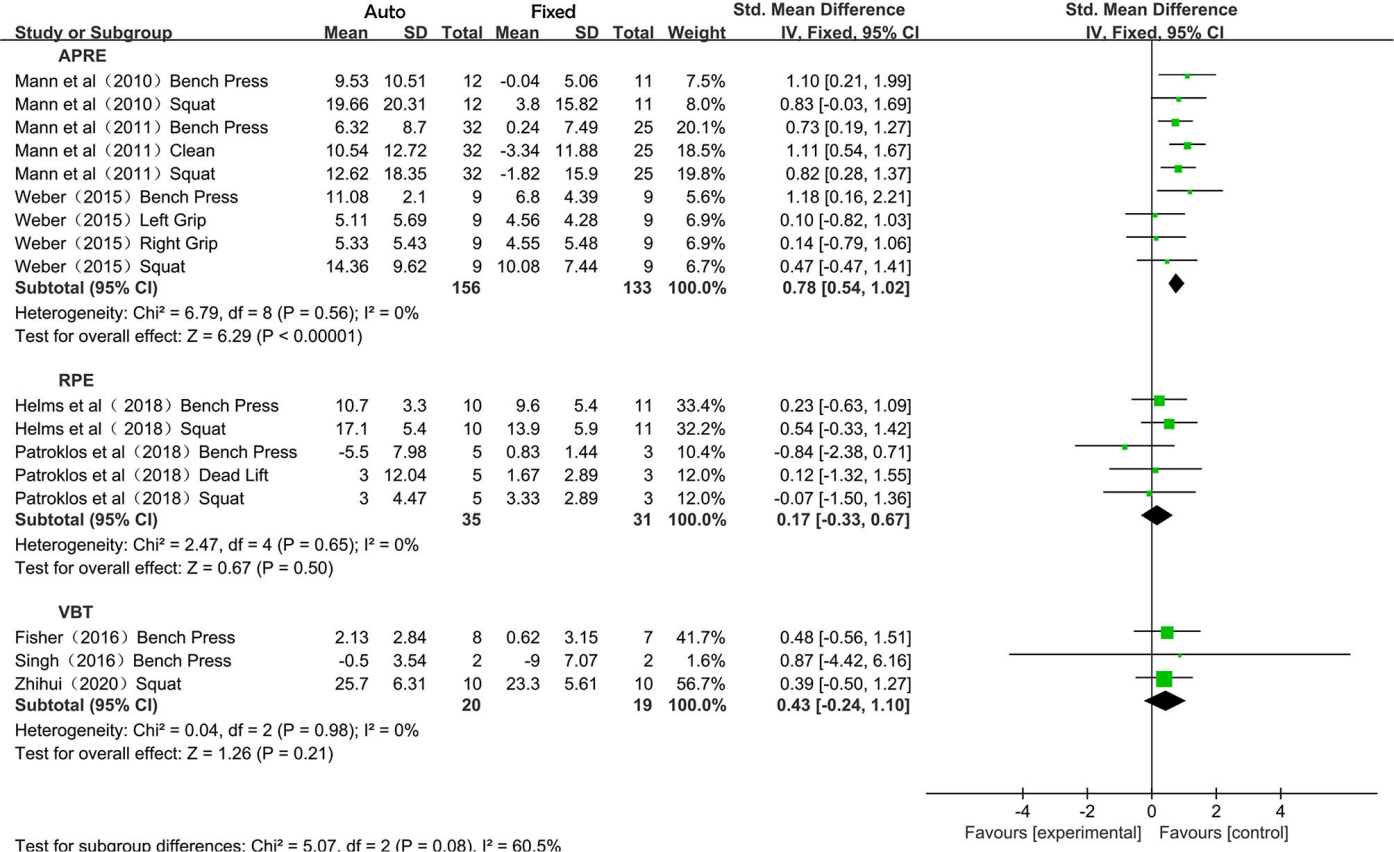
Forest plot referred to the effects of different auto-regulation programs (APRE, RPE, and VBT) in maximum strength training.

Due to the different events in the training programs, the subgroup analysis was performed and presented in SMD to compare the difference between APRE, RPE, and VBT training programs (Figure 5). As no heterogeneity was observed in each of the subgroup synthesis (*I*^2^ = 0%), the fixed-effects model was therefore employed, and the overall effects of APRE, RPE, and VBT were 0.78 (95% CI 0.54–1.02; *P* < 0.05), 0.17 (95% CI −0.33–0.67; *P* = 0.50), and 0.43 (95% CI −0.24–1.10; *P* = 0.21), respectively. No statistically significant difference was found in overall effects between the three methods (*P* = 0.08).

### Sensitivity Analysis

Due to the varied retrieved data, both the fixed- and random-effects models were used in the present study. To check the reliability of the results, we switched the used models for each of the synthesis, and the significance of the differences remained in each of the comparisons. As heterogeneity was observed in the squat and bench press data, the leave-one-out method was used to identify the source of the heterogeneity. The results indicated that two studies (Mann et al., 2010; Patroklos et al., 2018) were responsible for the high heterogeneity in the bench press subgroup, and the *I*^2^ decreased from 62 to 27% after removing the two studies, and the difference was still statistically significant (MD = 3.27, 95% CI 1.18–5.37, *P* < 0.05). Likewise, after removing the two studies (Mann, 2011; Patroklos et al., 2018), heterogeneity was eliminated (*I*^2^ = 0%), and the difference remained significant when the fixed-effects model was employed (MD = 3.66, 95% CI 0.49–6.82, *P* < 0.05).

### Publication Bias

**Figure 6:**
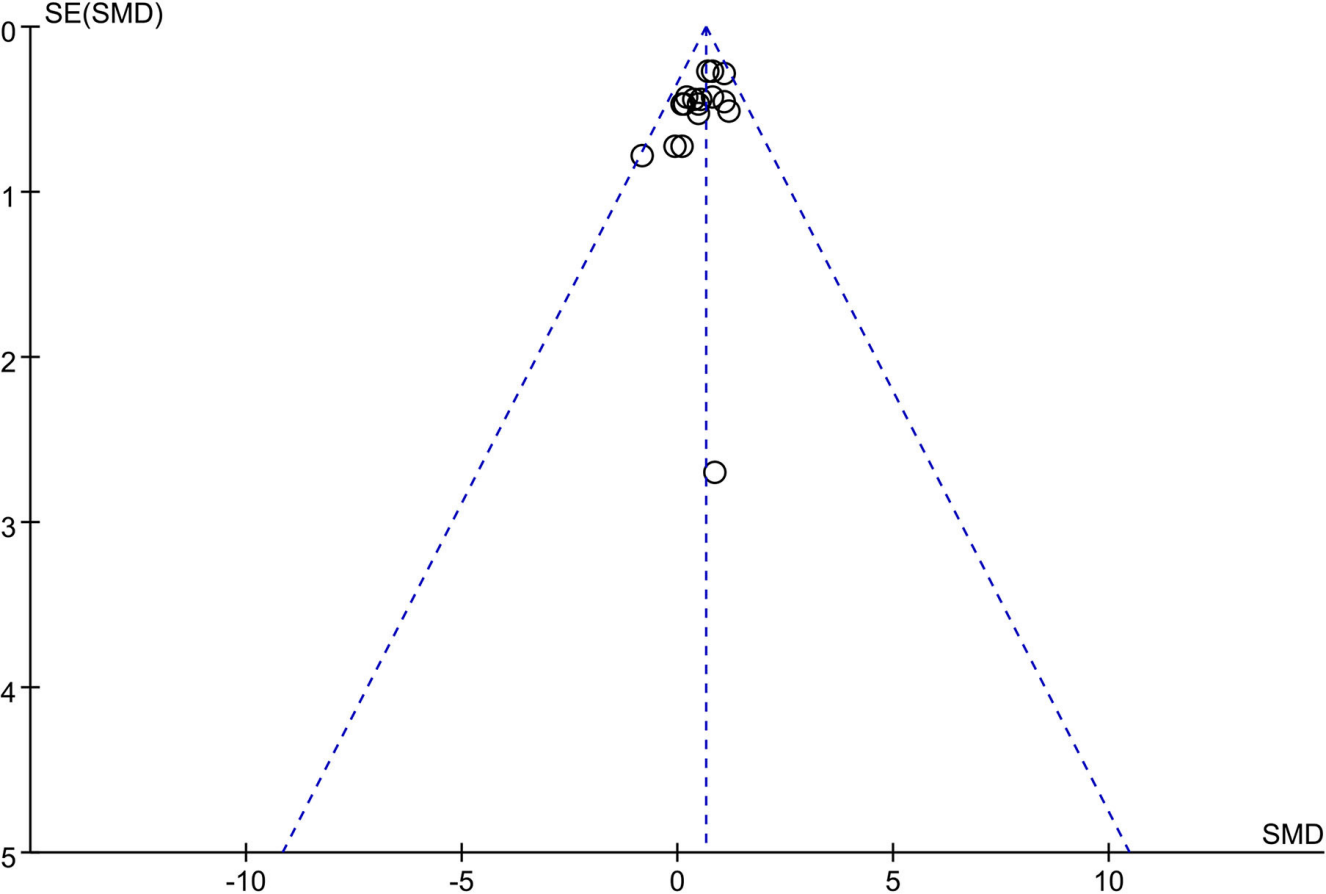
Funnel plots for a visual assessment of publication bias.

The funnel plot demonstrated more studies were distributed toward the left at the top of the graph, indicating a potential risk of bias (Figure 6). However, the plot was still close to symmetric, suggesting weak evidence of publication bias.

## Discussion

The present study aimed to compare the effects of auto-regulation and fixed-loading methods in maximum strength training, and checking the difference between different auto-regulation programs was another goal. As far as we know, our study was the first attempt to make such a comparison. The overall results indicated that the auto-regulation method was more effective in improving maximum strength than the fixed-loading method. As we stated in the *Introduction* section, considerable disagreements have remained in the previous studies (Mann et al., 2010; Mann, 2011; Fisher, 2016; Helms et al., 2018; Graham and Cleather, 2019; Peta, 2019; Dorrell et al., 2020). Based on the included studies, those controversial results might partly result from the different sample sizes. As the interventions disturbed the original training arrangement, these interventions may cause negative impacts on competitive results, especially for professional athletes, making it hard to recruit experienced athletes or conduct long-term interventions. Therefore, limited subjects were included in these studies, thus may lead to subtle differences. In the present study, the most significant results were reported in the study by Mann (2011), which had the largest sample size (57 athletes) among the included studies. In contrast, those with eight or fewer subjects only reported negative results (Fisher, 2016; Singh, 2016; Patroklos et al., 2018). In response, our meta-analysis included 166 experienced athletes from the studies with good or fair qualities, and provided strong evidence of using the auto-regulation method to enhance maximum strength in athletes.

Because the events, duration, and frequency of training were controlled in the treatment and control groups, the advantages of auto-regulation that we observed could be attributed to the different working loads, including various training intensities and volumes. In fact, some included studies and other related studies have reported that auto-regulation programs produced different working loads compared to the fixed-loading counterparts (Singh, 2016; Styles et al., 2016; Helms et al., 2018; Graham and Cleather, 2019; Dorrell et al., 2020). Theoretically, skeletal muscle growth and strength performance are regulated by neurogenic and myogenic factors (Rubinstein and Kelly, 1978; Kelly and Rubinstein, 1980), which are closely associated with the working load in training. Specifically, the different working loads can result in differential neuromuscular system adaption and growth hormone secretion, further affecting the performance and development of muscular strength. In general, our findings supported the theory in previous studies that the auto-regulation methods may provide more suitable working loads to maximize the training benefits (Eston et al., 1987; Naclerio et al., 2011; Scott et al., 2013; Dorrell, 2019), and also reduce risks in muscle damage and tissue injury that may result from exhaustive exercise (Suzuki et al., 2020).

Bench press and squat are the main training events in the included studies for quantitative synthesis, and the pooled results demonstrated that the auto-regulation methods increased the maximum strength for both training events. In the published literature, the bench press and squat are the core tests for athletes' fundamental strength, and play essential roles in athletic performances (Hoffman et al., 2007; Patrick et al., 2011), thus these results indicated the significance of the auto-regulation method in enhancing fundamental strength. In addition, competing athletes usually need integrated training to improve athletic performance, such as plyometrics training combined with strength training (Cherni et al., 2021). The auto-regulation method may provide flexible training arrangements for athletes to finish their training goals. For instance, there is evidence showing that athletes who resorted to the auto-regulation method for strength training had better performances in athletic performances, such as increased levels of agility and vertical jump (Styles et al., 2016; Arede et al., 2020; Zhihui, 2020). Considering that the included subjects were majored in various sports, including basketball (Dorrell et al., 2020), wrestling (Weber, 2015), and football (Mann et al., 2010; Mann, 2011), our findings further suggested the potential of the auto-regulation method in improving athletic performance for various sports.

In the previous studies on physical interventions, we found that a large number of training programs used 5–7 weeks to obtain significant results (Tesch et al., 2004; Ghigiarelli et al., 2009; McLeod et al., 2009; Genevois et al., 2014; Luebbers et al., 2014; Yan et al., 2016). Therefore, we divided the included studies into two groups, one with interventions of 5–7 weeks, and the other of 8–10 weeks. We found that the auto-regulation programs with shorter interventions (5–7 weeks) induced a significantly greater improvement than the fixed-loading counterparts. By comparison, those with longer interventions (8–10 weeks) induced a subtle difference (*P* = 0.05). Moreover, a significant difference was found between the two groups, indicating that the auto-regulation method might only contribute to short-term advantages. However, these results need to be further confirmed, because the longer interventions only ranged from 8 to 10 weeks due to the study design, and some other studies with longer interventions, such as an identified study of a 12-week intervention, were not included because of the invalid data (Graham and Cleather, 2019). Besides, fewer subjects participated in the longer interventions (81 vs. 130 subjects), which may affect the significance of the results. As we stated before, professional athletes may not participate in a time-consuming intervention, and also many subjects failed to complete the interventions (Helms et al., 2018; Patroklos et al., 2018), thus leading to limited results.

Among the AREP, RPR, and VBT programs, we found the APRE program more effective in improving the maximum strength compared to the fixed-loading method. However, a non-significant difference was found in the results of RPE and VBT programs. The reason for the differential results was still unknown, which might be associated with used measuring scales and the required equipment. The APRE program is regulated based on the completion reps, which does not require equipment and specific knowledge or experience, and therefore is easily accepted and well-applied. In contrast, the RPE program needs subjects to estimate their exertion precisely, which may cause difficulties in interventions. Some studies have reported that many subjects cannot estimate their exertion properly according to the measuring scale (Hackett et al., 2017; Steele et al., 2017; Keller et al., 2018), which may lead to some improper training (intensity and volume) during the intervention, and could be a potential reason for the non-significant difference. In the case of the VBT program, though the training is regulated based on the velocity that is unlikely limited by subjects' knowledge, the higher requirements in equipment and complex operations may cause inconvenience in training (Gomez-Piriz et al., 2013; Kimitake et al., 2015; Banyard et al., 2017; Orange et al., 2019a), which may further exert negative impacts on subjects' efforts in multiple interventions, and even mask the effects of auto-regulation. Moreover, in the present study, 53 subjects were involved in the APRE program, while only 15 and 20 subjects were involved in the RPE and VBT programs, respectively, and only the VBT program included female subjects, which may also partially explain the non-significant results.

On the other hand, though the RPE and VBT programs seemed no better than the fix-loading counterparts, they still showed potential in maximum strength training (Fisher, 2016; Helms et al., 2018; Patroklos et al., 2018; Zhihui, 2020). For instance, among those studies showing non-significant comparisons, Zhihui (2020) reported that both the VBT and fixed-loading programs induced improvements in maximum strength, and the increase obtained in the VBT program was statistically significant. Similarly, Helms et al. (2018) also reported that the maximum strength of subjects increased significantly from the baseline due to the RPE program. Taken together, these results indicated the effectiveness of the VBT and RPE programs in strength training.

With the intriguing findings in the present study, there are still some limitations. Overall, the auto-regulation method is a rising training method and widely commended, but its function in maximum strength training has not received sufficient concerns, which might be due to the difficulties in recruiting subjects and conducting long-term interventions. Therefore, only a few studies met the inclusion criteria of the present study, and our findings in subgroup analysis still need further validation. Besides, the present study only included experienced athletes, but whether the auto-regulation methods are suitable for sportspeople at different athletics levels is still unknown. Furthermore, to reduce the risk of injury in failed 1RM attempts and avoid invalid results due to inappropriate lifting strategies, many studies choose to evaluate the maximum strength via the 1RM formula (Niewiadomski et al., 2008; Grgic et al., 2020). However, the accuracy of evaluation can be affected by formula types, training items, completed reps, and subjects' characteristics (Wentworth and Abadie, 1998; Julio et al., 2012; DiStasio, 2014). In the included studies, only 49 (29.5%) subjects used the direct test, and they underwent different training programs and durations, making it hard to compare the differences between the two 1RM measurements. Nevertheless, the 1RM formula is still accepted as an effective and practical tool for maximum strength evaluation (Grgic et al., 2020), and future reviews may need to focus on the studies using the same formula and similar subjects to give more accurate results. In addition, only one included study recruited 15 female subjects, and they only participated in the VBT program, thus, we only evaluated the effects based on all subjects, and the gender difference was not analyzed, which may remain a future topic. Finally, the bench press and squat are the two training events involved in most studies, while other classical training, such as the deadlift and clean and jerk, was investigated only in one study (Patroklos et al., 2018); therefore, their effects are still inconclusive and remain to be investigated.

## Conclusions

The present systematical review and meta-analysis aimed to compare the differences in maximum strength improvement between the auto-regulation methods and fixed-loading methods. Our findings suggest that the auto-regulation method is more effective in improving maximum strength, which may help to improve the squat and bench press strength and further improve athletic performance, and athletes may largely benefit from the auto-regulation method by training of 5–7 weeks. Among the APRE, PRE, and VBT programs, the APRE may be the best option in the auto-regulation method, which is more effective and requires no equipment or training knowledge, thus may be of better potential in athlete training. Due to the limited available data, further investigation is warranted to better understand the advantages of different auto-regulation programs and figure out how maximum strength responds to various intervention duration interventions. Moreover, studies are needed to focus on female athletes and sportspeople at various athletics levels.

## Data Availability Statement

The original contributions presented in the study are included in the article/supplementary material, further inquiries can be directed to the corresponding authors.

## Author Contributions

XZ and HL wrote the manuscript, and SB joined in the discussion for paper inclusion and helped with the English editing. YL reviewed and helped revise the manuscript. YC and GZ supervised the project and amended the final version of the manuscript. All authors contributed to the article and approved the submitted version.

## Conflict of Interest

The authors declare that the research was conducted in the absence of any commercial or financial relationships that could be construed as a potential conflict of interest.
